# Monoclonal antibodies point to Achilles’ heel in picornavirus capsid

**DOI:** 10.1371/journal.pbio.3000232

**Published:** 2019-04-17

**Authors:** Mihnea Bostina

**Affiliations:** Department of Microbiology and Immunology and Otago Micro and Nano Imaging, University of Otago, Dunedin, New Zealand

## Abstract

Picornaviruses are small, icosahedral, nonenveloped, positive-sense, single-stranded RNA viruses that form one of the largest and most important viral families. Numerous Picornaviridae members pose serious health or agricultural threats, causing diseases such as poliomyelitis, hepatitis A, or foot-and-mouth disease. The antigenic characterization of picornavirus capsids plays an important role in understanding the mechanism of viral neutralization and the conformational changes associated with genome release, and it can point to regions which can be targeted by small-molecule compounds to be developed as antiviral inhibitors. In a recent study, Cao and colleagues applied this strategy to hepatitis A virus (HAV) and used cryo-electron microscopy (cryo-EM) to characterize a well-conserved antigenic site recognized by several monoclonal antibodies. They further used computational approaches to identify a small-molecule drug with a strong inhibitory effect on cell attachment.

## Abstract 2

This Primer explores the implications of a recent structural characterization of picornavirus capsid antigenicity, which points to regions that can be targeted by small-molecule antiviral inhibitors.

When Paris pierced Achille’s heel with an arrow, it was not just a lucky shot. It was a process which demanded 10 years to accomplish, the help of a god, and a detailed knowledge of the structural weakness of the enemy. The success stemmed from recognizing the exact position to be used as a target, a fact that was not apparent just by looking at the warrior. Similarly, structural information of a virus weakness cannot be inferred solely from virion architecture but has to be corroborated with other structural clues.

Virions are under constant attack either from environmental factors or from the immune system defense of the infected host. Monoclonal antibodies (mAbs) are weapons of high specificity binding to precise epitopes, forcing the virus to mutate surface residues in order to escape recognition. This process is much more complicated for icosahedral viruses because their capsid proteins fulfill simultaneously multiple roles. They must protect the viral genome, recognize the cellular receptor, assist viral uncoating, and assemble into newly formed virions. This number of constraints makes particularly relevant the study of epitopes on the external surface of the capsid targeted by antibodies.

Picornaviruses are small, icosahedral, nonenveloped, positive-sense, single-stranded RNA viruses that form one of the largest and most important viral families. Numerous of its members pose serious health or agricultural threats, causing diseases such as poliomyelitis, hepatitis A, or foot-and-mouth disease. Currently, the Picornaviridae family comprises over 80 species divided in 35 genera [1] and is undergoing a constant expansion with many other potential members being sequenced and waiting to be classified. A typical picornavirus has a genome between 7,200 and 10,000 nucleotides translated into a single polypeptide chain processed into 3 segments. The first segment, P1, is responsible for the structural proteins, whereas the P2 and P3 segments are further cleaved usually into 6 nonstructural proteins involved in RNA replication [2,3].

## Picornavirus structure

**Fig 1:**
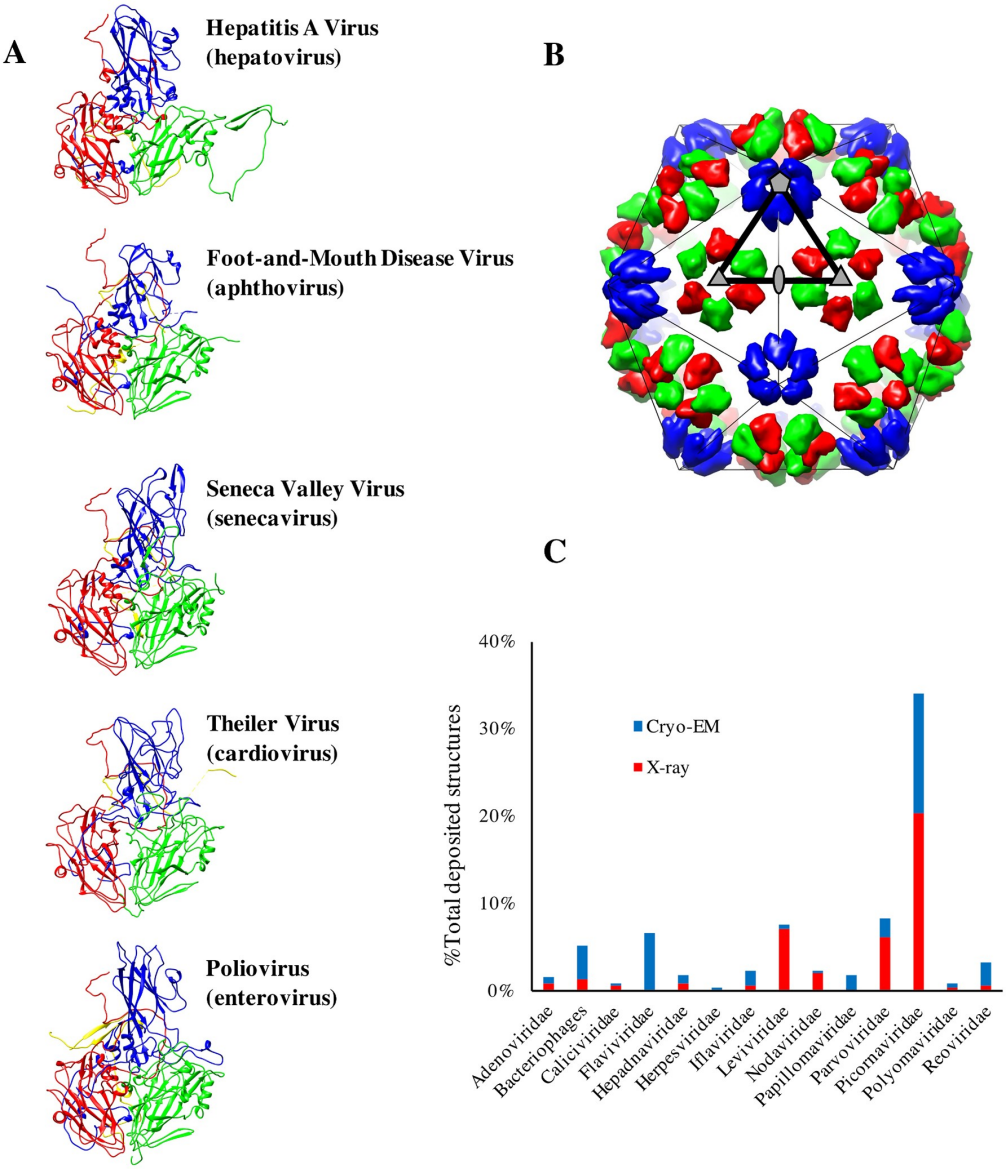
Picornavirus architectures. (A) Picornavirus capsids are assembled from 60 copies of 4 capsid proteins: VP1 (blue), VP2 (green), VP3 (red), and VP4 (yellow). The protomer structure of representative picornaviruses genera show a common fold. The PDB accession numbers of HAV, foot-and-mouth disease virus, Seneca Valley virus, theiler virus, and poliovirus are 4QPI, 4GH4, 3CJI, 1TME, and 2PLV, respectively. (B) The 3 large capsid proteins VP1, VP2, and VP3 have a beta-barrel fold that assemble in a similar distribution of 180 beta-barrels common in all picornaviruses. The overall morphology of the capsids is dictated by the loops connecting the beta strands and by the length and conformations of the N- and C-termini. The icosahedral geometry is delineated by a thin line, and the asymmetric unit is indicated by a thick black line, with the 5-, 3-, and 2-fold axes of symmetry marked by a pentagon, a triangle, and an ellipse, respectively. (C) Distribution of structures from representative families of icosahedral viruses solved via X-ray crystallography or cryo-EM methods. cryo-EM, cryo-electron microscopy; HAV, hepatitis A virus.

The P1 segment is proteolytically cleaved to form protomers consisting of 3 large proteins—VP1, VP3, and VP0—that can be further cleaved into VP2 and VP4. Protomers form stable pentamers with VP1s arranged parallel to the 5-fold axis. At their turn, 12 pentamers assemble into a complete icosahedral capsid with VP1 distributed around the 5-fold axis, whereas VP2 and VP3 alternate around the 3-fold axis. VP4 is located at the interior of the capsid, and in many picornaviruses, the cleavage from its precursor is associated with genome encapsidation. The 3 major capsid proteins—VP1, VP2, and VP3—have an identical fold consisting of an 8-strand flattened beta-barrel (Fig 1a). Their arrangement describes a capsid with an identical distribution of 180 beta-barrels common to all picornaviruses (Fig 1b). Individual differences are dictated by the loops connecting the beta-strands and by the length and conformation of the N- and C- termini (Fig 1c) that govern the general appearance for each species [4–7]. The complicated geometry of invading arms and loops from VP1, VP2, and VP3 together with the reinforcement provided by VP4 at the interior of the capsid generate a stable capsid capable to harbor the densely packed RNA genome. Nevertheless, this state cannot be too rigid: either receptor binding and/or changes in pH have to be able to trigger conformational changes facilitating the delivery of the genome to the cellular cytoplasm where the viral replication takes place.

Recent advances in cryo-electron microscopy (cryo-EM) have enabled a spectacular increase in reported picornavirus structures adding to the already rich amount of information gathered by X-ray crystallography. Picornaviruses are the best structurally characterized family with over 260 structures [8], representing more than one-third of the atomic coordinates of icosahedral viruses currently deposited in databases (Fig 1c). These structures document a wide spectrum of capsid conformers corresponding to different stages of picornaviral lifecycle, including isolated capsids, capsids engaging the cellular receptor, capsids undergoing conformational changes associated with genome release, or capsids decorated with fragments of mAbs.

Picornaviruses recognize several classes of receptors such as members of the immunoglobulin-like family, the low-density lipoprotein receptor family, and the integrin family of cell adhesion molecules [3]. Following receptor binding, picornaviruses release their genome according to different mechanisms. For enteroviruses, the process was shown to involve a conformation change that widens a gap along the 2-fold axis allowing the exit of viral RNA [9]. In the case of cardio-, aphtho-, and senecaviruses, the capsid dissociates into pentamers releasing the viral RNA in the host cell. Structural basis of receptor recognition was elucidated in detail in the case of several picornavirus genera [10–13] offering a wealth of information on the role of specific residues. Nevertheless, when such insights are unavailable, valuable information can be obtained by studying the capsid interaction with neutralizing antibodies.

## Antigenicity of picornavirus capsid

Depending on the geometry of antibody–capsid contact, several mechanisms of viral neutralization can be inferred. First, antibodies can cross-link different particles causing aggregation and thus impeding the uptake by the cell. Secondly, the antibody can compete with the receptor for viral interaction, occluding the receptor binding site and thus preventing cell attachment. Thirdly, the antibody binding can trigger a conformational change that renders the virus incapable to progress to a successful infection. Finally, the binding can arrest the virion in a rigid conformation, making it impossible for the suite of capsid rearrangements necessary for genome release.

Assuming a single mechanism of virus neutralization by mAbs could be a dangerous simplification. The same picornavirus serotype can be neutralized by different mechanisms [14]. Structural studies are generally conducted using fragment of antigen binding (Fab) fragments and extrapolating the mechanism to full mAbs might be problematic. In many cases, the Fab fragments fail to offer effective neutralization despite binding tight to the capsid, whereas the full mAbs can successfully cross-link adjacent epitopes on viral capsid, blocking further conformational changes. The number of Fabs bound to a single capsid can also vary. A common case is the total occupancy with the binding sites strictly obeying the icosahedral symmetry. Nevertheless, it was shown in several situations that steric clashes can limit the number of Fab fragments capable of occupying equivalent positions on the capsid surface [15]. Another case is represented by Fabs binding at the interface of 2 pentamers rigidizing the capsid in a closed conformation [16]. In principle, this would prevent the genome release. However, the coexistence of mutations with wild-type variants could still salvage the infection. In this scenario, different combinations of pentamers, with different residues in equivalent position will assemble into a mosaic capsid in which the formation of the recognizable epitope by mAbs will be partially avoided. An alternative situation was observed in enteroviruses in which antibody binding can neutralize infection by either partially destabilizing the capsid [17] or triggering the premature release of viral genome [18].

Antibodies blocking receptor binding recognize specific epitopes and overlap at least partially with the footprint of receptors on viral capsid. In the absence of structural information on receptor–capsid interaction, studies of different mAbs can delineate capsid residues essential for receptor recognition. A set of antibodies could recognize the same patch on a viral capsid and point to an area of high antigenicity. At the same time, the epitope recognized by a receptor on the virus surface could also tolerate mutations, and antigenic variability can be observed even within the receptor-binding footprint [13]. In genera characterized by genotypic abundance such as enteroviruses, neutralizing epitopes could be conserved, and cross-neutralization can occur between serotypes [19]. The opposite is also possible, antigenic variation being present in isolates belonging to the same genotype [14].

## Hepatitis A virus

Hepatitis A virus (HAV) holds a special place in the Picornaviridae family due to several characteristics, including: the unique architecture of the capsid proteins, the extreme robustness of the virion, the capacity to use cellular membranes to escape antibody recognition, and the existence of a single serotype dictated by codon usage constraints.

**Fig 2:**
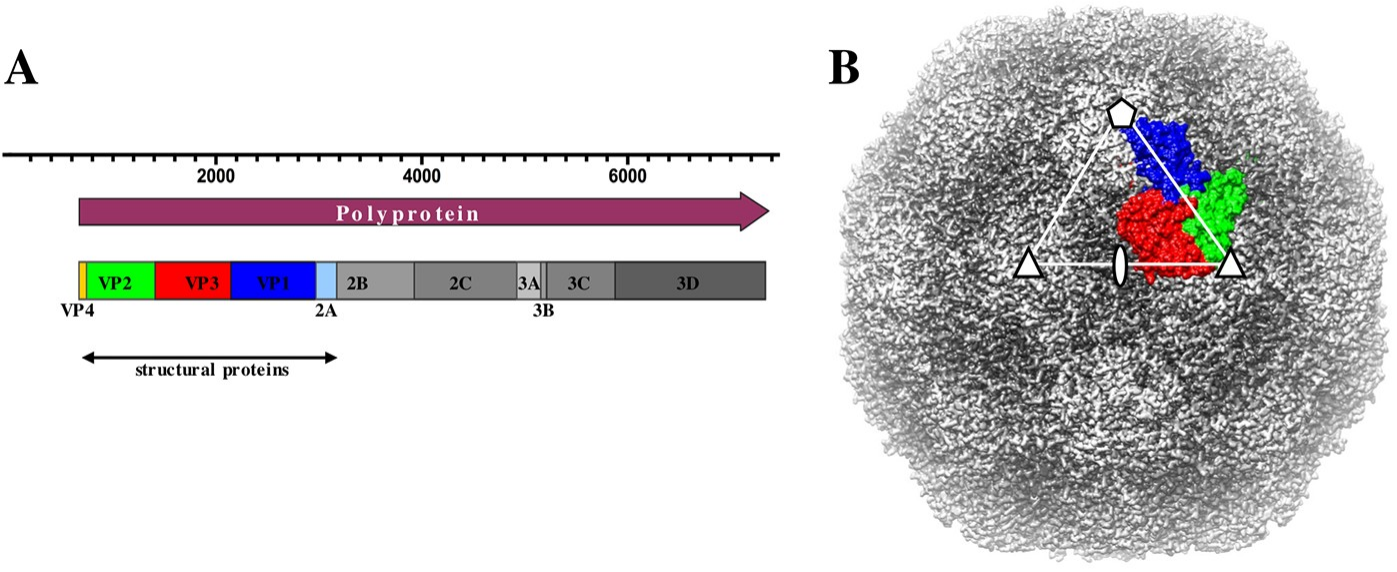
HAV genome and capsid structure. (A) Organization of the HAV genome: the structural proteins VP1, VP2, VP3, and VP4 are colored in blue, green, red, and yellow, respectively. (B) Surface rendering of HAV capsid with individual proteins belonging to a single protomer colored according to the same convention. The asymmetric unit and the axes of symmetry are indicated. HAV, hepatitis A virus.

Phylogenetic analysis based on capsid structure comparison locates HAV distant from other picornavirus genera and in close genetic proximity to insect picorna-like viruses [7]. This eccentricity is evident when considering the capsid geometry, in which VP2 has a long N-terminal domain that extends along the interpentamer boundary and adds an extra strand to the VP2 beta-barrel from the neighboring pentamer [7]. Another unique trait of HAV capsid is the processing of the long C-terminal extension of VP1 protein essential for capsid assembly that is cleaved in the mature virion. These morphological alternatives are unique to HAV, but they do not entail significant changes in the overall genome structure and appearance of the HAV capsid, which retains the similarity to other picornaviruses (Fig 2).

Another particularity of the HAV capsid is its unusual rigidity, which makes it capable to tolerate extreme conditions such as pH = 2 and a high temperature of 80 °C. This behavior is explained by strong residue interactions around the 2-fold axis [7], a region shown to perform a major conformational change that permits the RNA release in enteroviruses [9].

Dramatic remodelling of membranes inside the infected cells is a general characteristic for all RNA viruses. Unexpectedly, in the case of HAV, it was shown that the virion is capable of hijacking cellular membranes and escaping into extracellular space enclosed in cellular vesicles. Such a camouflage allows virions to avoid the recognition by neutralizing antibodies and spread inside the healthy liver tissue [20] in a manner reminiscent of enveloped viruses. This mechanism of viral egress was later observed in other picornaviruses [21], blurring the clear distinction between enveloped and nonenveloped viruses.

Despite there being worldwide spread and it probably being present since antiquity, HAV has a single serotype, an atypical case within the Picornaviridae family. Icosahedral viruses can tolerate mutations at the exterior of the capsid that translate into antigenic variability even in the absence of immunogenic pressure. Due to the lack of structural constraints, the exterior of the capsid is expected to have a higher rate of amino acid replacement compared with regions located inside the capsid. Unusually, in the case of HAV, the same low mutation rate was observed for both inside and outside the capsid. It was shown that rare codon usage has a strong limiting effect on HAV genotypic variation [22]. In general, an amino acid can be encoded by each of the equivalent codons without any effect on the translated peptide chain. However, the use of codons present less frequent in the host cell will cause the ribosome to stall until the desired codon becomes accessible, thus impeding the speed of genome translation. The strategy of using rare codons assures that the translated peptide chain has the necessary time for proper folding. This is especially relevant in the case of residues located around or inside the antigenic regions where there is a strong evolutionary pressure to keep them unchanged in order to not disturb the balance between the rate of translation and the protein folding.

The highly conserved antigenic stability of the HAV capsid has enabled the manufacture of a very efficient vaccine. Nevertheless, currently only 16 countries use the vaccine for routine immunization of children [23]. As a result, the burden of HAV infections is still significant. More than 1.5 million people are annually infected with HAV, the infections being mostly associated with poor sanitation, water quality, housing density, and income [23,24]. The danger of HAV epidemics is inversely correlated with the endemicity levels, a phenomenon known as the “paradox of HAV risk.” HAV is generally asymptomatic in young children whereas in adults it causes the typical disease and its related complications. Therefore, a high endemicity level of HAV is associated with infection at an early age and results in a high level of immunity in the adult population. On the other hand, in areas with low infection rates, the majority of the adult population lacks immunity, and there is an increase in the median age at which infection occurs causing more severe symptoms [24]. Together with immunization efforts, especially in countries with high and intermediate endemicity, the development of antiviral drugs will be useful for treating fulminant infections. A better characterisation of the HAV antigenic profile could offer insights into the neutralization mechanism and suggest structure-based rationale for the design of efficient antiviral inhibitors.

Even if the detailed structural characterization of the HAV capsid is available [7], the external morphology does not offer any indication on the possible receptor binding site. Recently it was shown that the T-cell immunoglobulin and mucin domain 1—for a long time believed to act as the HAV receptor—is not essential for the cell entry or viral replication of both enveloped and nonenveloped virions [25]. This situation made the antibody–capsid investigations an attractive alternative for obtaining structural information. A previous cryo-EM study revealed the mechanism used by a potent neutralizing antibody to block the HAV attachment to the host cell [26].

In an article published in this issue of *PLOS Biology* [27], the same group investigates a further series of mAbs that potently neutralize HAV at nM concentrations [27]. Their cryo-EM maps reached a resolution that permitted to build atomic models for all the Fab–capsid complexes. The series of structures show almost identical features and demarcate a single well-defined patch on the capsid surface for all the mAb binding sites. The recognized epitope confirms the antigenic site previously reported. The authors argue that this architecture presents unique features in picornaviruses. All antibodies make an extended contact with VP3 but also bind several residues of VP2 from a neighboring pentamer. This interaction outlines an antigenic area that covers inter-pentamer contacts along the two-fold axis. The residues constituting the epitope are highly conserved in all HAV genotypes, and the variation rate for the epitope is lower than for the whole capsid, indicating a key structural role.

The results from Cao and colleagues highlight a few important points with broad relevance for picornavirus mAbs neutralization. First, it shows that epitopes described by neutralizing antibodies suggest good targets for drug design. Secondly, the binding strength is a good predictor of antiviral potency. Finally, the equivalence of the Fab–capsid interaction for all the investigated antibodies together with sequence analysis of the conserved viral epitope indicate that the mAbs are likely to neutralize all HAV genotypes.

In the next step, the authors observed that the essential residues of the antigenic site are distributed along a depression region that was proposed to have a critical role in receptor binding. This structural role recommends this location as a target for small-molecule antivirals. They chose 4 residues in the mAbs’ heavy chains that have a major contribution to the antibody–capsid contact and used them as a model for an in silico docking screening of a large drug database. The best fit identified golvatinib as a potential candidate. A cell-based antiviral assay confirmed that indeed golvatinib is capable to block HAV infection with high efficacy.

The approach used by Cao and colleagues proves the importance of possessing a detailed structural characterization of the antigenic profile of a virus capsid and the power of the structure-based design of small-molecule inhibitors. Remarkably, the combination of structural and computational techniques was capable to deliver a valid single small-molecule drug in a single round of design. In recent years, cryo-EM has proved itself to be a powerful method to characterize virus–antibody interactions. Such analysis can reveal highly conserved antigenic sites pinpointing epitopes with a key structural roles and thus constituting good antiviral targets. This strategy is an attractive road for the discovery of antiviral drugs against other pathogenic viruses.

## Abbreviations

cryo-EM: cryo-electron microscopy
Fab: fragment of antigen binding
HAV: hepatitis A virus
mAb: monoclonal antibody
